# UASOL, a large-scale high-resolution outdoor stereo dataset

**DOI:** 10.1038/s41597-019-0168-5

**Published:** 2019-08-29

**Authors:** Zuria Bauer, Francisco Gomez-Donoso, Edmanuel Cruz, Sergio Orts-Escolano, Miguel Cazorla

**Affiliations:** Institute for Computer Research, University of Alicante, P.O. Box 99, 03080 Alicante, Spain

**Keywords:** Databases, Electrical and electronic engineering

## Abstract

In this paper, we propose a new dataset for outdoor depth estimation from single and stereo RGB images. The dataset was acquired from the point of view of a pedestrian. Currently, the most novel approaches take advantage of deep learning-based techniques, which have proven to outperform traditional state-of-the-art computer vision methods. Nonetheless, these methods require large amounts of reliable ground-truth data. Despite there already existing several datasets that could be used for depth estimation, almost none of them are outdoor-oriented from an egocentric point of view. Our dataset introduces a large number of high-definition pairs of color frames and corresponding depth maps from a human perspective. In addition, the proposed dataset also features human interaction and great variability of data, as shown in this work.

## Abstract (summary)

**Table:** 

Design Type(s)	modeling and simulation objective • image creation and editing objective • database creation objective
Measurement Type(s)	image
Technology Type(s)	digital camera
Factor Type(s)	geographic location
Sample Characteristic(s)	Alicante • anthropogenic habitat

Machine-accessible metadata file describing the reported data (ISA-Tab format)

## Background & Summary

The use of supervised deep learning algorithms has created the need for massive amounts of information to achieve their great generalization capabilities. However, focusing on depth prediction, the released datasets that provide both color images and corresponding depth maps are, so far, either synthetic^1,2^ or yield a reduced number of samples^3,4^. Synthetic data is generally unrealistic due to the quality of the data, or because of the composition of the scenes, so that in many cases the resultant model requires fine-tuning using real world data^1^. Real-world datasets with insufficient numbers of samples limit the generalization capabilities of the algorithms, which makes them unusable for real-world deployment.

Only four of the datasets shown in Table 1 are outdoor datasets. Below is a review of each of them, highlighting the most important aspects with a special focus on the following features: size of the dataset, resolution, outdoor or indoor and the viewpoint of the recording.

**Table 1:** Review of the main elements of the most popular RGB-D dataset.

Dataset	Scale		Resolution	Modality			Outdoor	Video
	Frames	Scenes		RGB	Depth	Point Cloud		
SCENENET RGB-D^1^	5 M	57	320 × 240	✓	✓	✗	✗	✓
MATTERPORT 3D^24^	200 k	90	1280 × 1024	✓	✓	✓	✗	✓
STANFORD 2D-3D^25^	70 k	270	1080 × 1080	✓	✓	✓	✗	✓
SCENE NN^26^	2 k–10 k each scene	100	640 × 480	✓	✓	✗	✗	✓
MICROSOFT RGB-D^27^	500–1 k each seq.	7	640 × 480	✓	✓	✗	✗	✓
VIDRILO^28^	22 k	10	640 × 480	✓	✗	✓	✗	✓
SUN RGBD^29^	10 k	270	1080 × 1080	✓	✓	✓	✗	✗
NYU-D V2^30^	1.5 k	464	640 × 480	✓	✓	✗	✗	✓
B3DO^31^	849	75	1080 × 1080	✓	✓	✗	✗	✗
SYNTHIA^2^	200 k		960 × 720	✓	✓	✗	✓	✗
KITTI^5^	1.6 k	400	1392 × 512	✓	✓	✓	✓	✗
ETH3D^4^	898	25		✓	✓	✗	✓	✓
MAKE 3D^3^	534		1280 × 1024	✓	✓	✗	✓	✓
Tanks and Temples^6^	147791	14	1600 × 1200	✓	✓	✓	✗	✓
Middlebury Dataset^7^	40 k	33	2964 × 1988	✓	✓	✗	✗	✗
**OURS**	**482736**	**33**	2208 × 1242	✓	✓	✗	✓	✓

The important features for us are scale (number of frames and the number of different layouts or different scenes of the dataset), resolution (in pixel), modality (the data provided by the dataset) and whether it is an outdoor dataset.

SYNTHIA^2^ or The SYNTHetic collection of Imagery and Annotations consists of a collection of photo-realistic frames rendered from a virtual city. There are two main problems with this dataset. The first is that it is a synthetic dataset, meaning that the frames are not photorealistic, with the subsequent problem of testing the system in real conditions. The second problem is that the dataset is centered on the vision of a car driving in the street, while, in our case, we need the point of view of the pedestrians.

The KITTI dataset^5^ provides the RGB (stereo pair) and depth maps of 400 different layouts having a total of 1.6 k frames of roads from the city of Karlsruhe (Germany). This dataset is outdoor, so it fulfills one of our main requisites. The only problem of this dataset is that it was captured from the perspective of a car, so the main view is from the road.

The ETH3D dataset^4^ includes 534 RGB-D frames divided into 25 scenes. The ground-truth was taken with a highly accurate 3D laser scanner. This dataset provides outdoor data. Of the 25 scenes, only 9 are outdoor which significantly decreases the number of images.

Tanks and Temples^6^ includes 147791 RGB-D frames in 14 different scenes. The ground-truth data was captured using an industrial laser scanner which adds precision to the data. It only provides static scenes with no interaction, which leads to the scenes provided are being mostly objects. They provide only 6 scenes with complete rooms, which are not ordinary scenes and therefore could not provide good generalizability to the trained models.

The Middlebury Dataset^7^ provides 33 scenes, each filmed from two different exposures. All of the scenes provided are indoor, mainly focused on objects. The Middlebury dataset provides different lighting conditions for each scene, but as mentioned, theseare indoor static scenes. In addition, the amount of data is not enough to correctly train a more complex deep learning algorithm.

Finally, the Make3D dataset^3^ is outdoor and taken from the perspective of a pedestrian. The different scenes provide human interaction and also different types of paths and roads a pedestrian could use. It only contains 534 frames, so the scale of this dataset is the smallest of all those reviewed.

The dataset presented in this paper is UASOL ^8^: A Large-scale High-resolution Outdoor Stereo Dataset. It was created at the University of Alicante and consists of an RGB-D stereo dataset, which provides 33 different scenes, each with between 2 k and 10 k frames. The frames show different paths from the perspective of a pedestrian, including sidewalks, trails, roads, etc. The images were extracted from video files with 15 fps at HD2K resolution with a size of 2280 × 1282 pixels. Another important feature is the scale of the dataset. It provides about 160902 frames, thus yielding sufficient data to be able to train a deep learning network. The dataset provides a GPS geolocalization tag for each second of the sequences and reflects different climatological conditions. It also involved up to 4 different persons filming the dataset at different moments of the day. These features lend the dataset high variability, which will challenge the generalization capabilities of the algorithms (see Technical Validation section).

## Methods

In this section, we explain the methodology used to produce the data. We also describe the capture setup, the techniques we applied to generate the depth maps and the algorithms used to provide data for the technical evaluation.

### Overview

The proposed dataset is intended to be used for a number of different goals. For instance, stereo triangulation, structure from motion and depth estimation from monocular frames are problems that can also be benchmarked with UASOL.

**Fig. 1:**
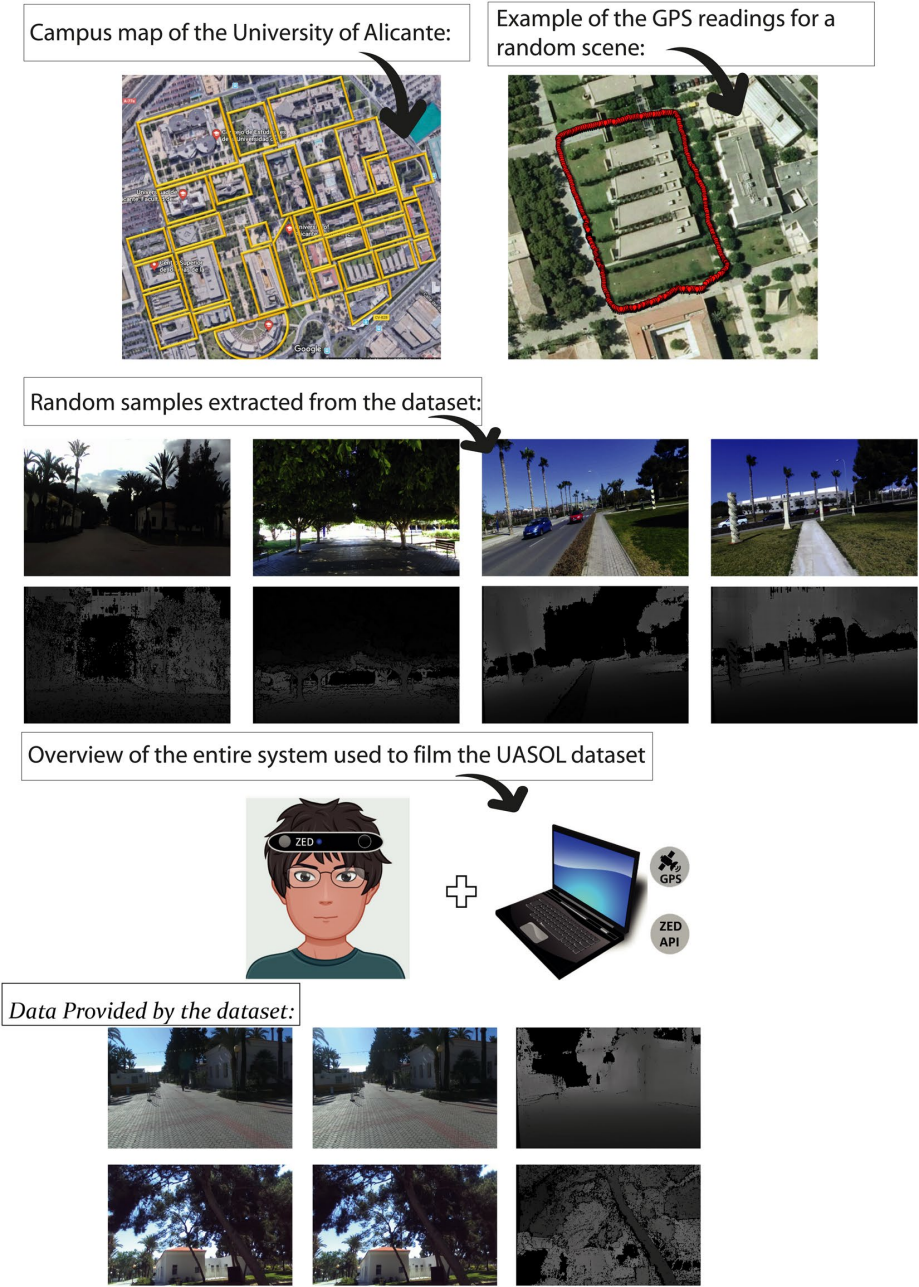
General overview of some of the informations and components of the UASOL dataset.

The dataset is structured in sequences. A sequence is composed of a set of frames. A frame comprises two images provided by a stereo camera, a depth map and a GPS tag. Each sequence of the dataset depicts the surroundings of a particular building on the University of Alicante campus, as depicted in Fig. 1.

### Capture setup

Two main hardware devices were used for the creation of the dataset. First, the images and the depth maps were captured by a ZED camera. The ZED is a 2 K stereo device with dual 4 MP RGB sensors, a 110° field of view and f/2.0 aperture. It supports a number of different resolutions and framerates. In order to provide better image quality, we set the resolution to the maximum, namely 2208 × 1242 resolution. This setting limits the framerate to 15 fps. This camera is able to sense depth values ranging between 0.7 and 20 meters. This range is limited by the baseline between the two cameras featured in the device.

The second hardware element is the GlobalSat GPS device. This device is able to provide GPS localization with an error below 3 meters. In addition, the GPS data is queried once per second, which is the maximum query rate of this device.

The ZED camera is attached to a strap worn by the subject which is capturing the sequence. This is done in order to provide an egocentric point of view. The ZED camera and the GPS are wired to a notebook that records the data.

In total, there are 643608 data samples distributed in 33 sequences covering the outdoor surroundings of 38 buildings in the dataset. This dataset features around 321804 RGB images and the same number of depth maps. Random samples of the dataset are depicted in Fig. 1. It is also included around 10599 GPS tags. We also provide positional tracking for each frame and calibration data for the camera. In addition, full ZED recordings are provided in SVO format in order to allow every user of the dataset to extract and process as they wish, using the ZED SDK and custom algorithms. We also provide some sample scripts to extract different types of data to be used as a stub for custom implementations.

### Procedure

This section illustrates the procedure followed to record the dataset. It is divided into the following subsections: First, an image acquisition and quality section in included, where we explain the setup used to record the sequences that comprise this dataset. There then, follows a section where the accuracy of the depth maps generated by the ZED Camera is tested. Next is a section where the offline processing of the dataset is detailed, explaining the stereo algorithm used to obtain the depth data. Following this section, we detail the different algorithms used for the data validation. The final section addresses the code availability, where we explain the scripts used for the data generation and some other utilities we released alongside the dataset itself.

### Data acquisition

As mentioned, the dataset was acquired using the ZED Camera. This device was installed on the forehead of different users, giving the data realistic features, such as vibration or twisted frames, from real non-static humans. The ZED camera was connected to a laptop with the ZED API installed to film the different sequences and store them in custom SVO files. The laptop also features the GPS device and this type of data was collected at the same time. Each GPS location is tagged by the systems clock timestamp. An overview of the entire system is shown in Fig. 1.

After each video sequence and its GPS data was stored, we used the ZED API to extract the information from the videos. The raw SVO files include all the features recorded for each sequence; when these are reproduced, it simulates the recording moment allowing access to all the attributes provided. The proposed dataset includes each data source of the ZED API. Thus, for each frame of a sequence, we provide the stereo pair, the estimated depth map by the ZED algorithm, the estimated depth maps by a novel deep-learning based stereo method, the camera information, a timestamp, the positional tracking of the camera and the GPS tag.

All this information was packed into a manifest file per sequence. We decided to distribute the manifests in JSON files because it allows a variety of programming languages and applications to be easily stored and loaded.

### Accuracy of the ZED camera

One of the main goals of the dataset is to provide accurate depth maps for training deepl learning based algorithms, for instance, depth estimation from monocular RGB images. Although many 3D cameras exist, a stereo device was chosen over structured light-based devices like Kinect v1 because the latter cannot operate in outdoor environments, as the projected IR pattern is lost due to the ambient light. Time-of-Flight (ToF) cameras, like the Kinect v2, were also discarded because of their poor performance in outdoor environments, as stated in^9^. Another key point for the camera choice was the work in^10^, where a detailed survey of Depth Data Error Estimation was carried out in the three most commonly used RGB-D Sensors: Zed Camera, Kinect v1 and Kinect v2. As the paper concludes (section 5.3), for distances greater than 3.5 m, the ZED camera obtains data with the lowest depth RMS error, which makes this depth sensor perfect for our dataset.

Consequently, we adopted the ZED camera, which is stereo-based. The accuracy is thoroughly tested in^11,12^. Furthermore, in^10^, the ZED camera was found to be more accurate over 3 meters than structured light based sensors like Kinect v1 and v2.

These works conclude that the ZED camera is definitely more suitable for outdoor environments and provides higher quality ground-truth on longer ranges. The key features of the ZED camera are shown in Table 2.

**Table 2:** Review of the main features of the ZED camera.

Features	
Size (mm)	175 × 30 × 33
Weight (g)	159
Image and Depth Resolution (pixels)	HD2K: 2208 × 1242 (15FPS), HD1080: 1920 × 1080 (30, 15FPS), HD720: 1280 × 720 (60, 30, 15FPS), WVGA: 672 × 376 (100, 60, 30, 15FPS)
Depth	Range (m): 1–20, Format (bits): 32 m Baseline (mm): 120
Lens	FoV: 110, *f*/2.0 aperture
Sensors	Size: 1/3, Format: 19:9, Pixel Size: 2-u pixels
Connectivity	USB 3.0 (5 V/380 mA), 0 C to + 45 C
SDK System	Windows or Linux, Dual-core 2.3 GHz, 4 GB RAM, Nvidia GPU

The ZED camera provides good results in outdoor environments. However, the depth error increases at greater distances, as shown in the Supplementary Material Fig. 1.

The depth error is modeled after the following equation:

$${e}_{zp}(pixel)=\frac{{z}^{2}\cdot {a}_{d}}{B\cdot f}$$

$${e}_{z}(mm)={e}_{zp}\cdot {p}_{size}$$

Where:

- *z* = *distance* (*mm*)
- *a*_*d*_ = *disparityaccuracy* (*pixel*) = *e*_*c*_ · 2
- *e*_*c*_ = *calibrationerror* (*pixel*) = 0.2
- *B* = *baselinecte* (*mm*) = 120
- *f* = *focallength* (*pixels*) = 1400
- *p*_*size*_ = *pixelsize* (*mm*) = 0.002

The expected errors for distances between 0 and 20 meters are shown in the Supplementary Material Fig. 1. scaled y ticks = false.

### Depth maps ground-truth: Semi-global matching and GC-Net

The ZED camera reproduces the way our binocular vision works. This sensor features two cameras separated by a baseline of 12 cm. The sensor is able to capture high-resolution 3D video and estimate depth maps and motion by comparing the displacement of pixels between the left and right images. The algorithm used to achieve the depth estimation is an implementation of the Semi-Global Matching (SGBM) algorithm^13^. The Semi-Global Matching method calculates the matching cost hierarchically based on Mutual Information. It uses this to compensate for the radiometric differences of the input images. The pixelwise matching used is supported by a smoothness constraint usually expressed as a global cost function. SGBM performs a fast approximation using pathwise optimizations from all directions. We used the Ultra mode setting to extract the depth maps, which removes all stereo matching points with a low confidence score. This settings adjustment allows the algorithm to better preserve the edges and the depth-accuracy while providing increased range.

Alongside the depth maps produced by the ZED SGBM algorithm, as a future work, we provide the depth maps computed by a novel deep-learning based stereo method named GC-Net^14^. This proposal predicts the disparity from a rectified pair of stereo images, leveraging knowledge of the geometry to form a cost volume using deep feature representations. The architecture learns to incorporate contextual information using 3-D convolutions over this volume. Disparity values are regressed from the cost volume using a proposed differentiable soft argmin operation, which allows the model to be trained end-to-end, achieving sub-pixel accuracy with no additional post-processing or regularization. This approach sets a new state-of-the-art benchmark on the KITTI^15^ dataset, while being significantly faster than competing approaches.

The results are shown in the Technical Validation section.

### Algorithms for data validation

To perform the technical validation of this dataset, two different deep learning-based techniques were used. First, an object detector is used to count the number of objects of a different class per sequence. Then, a pixel-wise classifier is used to count the ratio of pixels that represents each class per sequence.

#### Object counting procedure

To count the existing elements in the different frames of the sequences we used an automatic object detection system called YOLOv3^16^, which detects several common outdoor object categories. YOLO is a convolutional neural networ-based (CNN) system. CNNs are supervised machine learning techniques that are modeled on the brain structure, comprising a network of learning units called neurons. These neurons learn how to convert input signals (e.g. picture of a cat) into corresponding output signals (e.g. the label “cat”). In the case of YOLO, it is able to simultaneously predict multiple bounding boxes and class probabilities for those boxes.

**Fig. 2:**
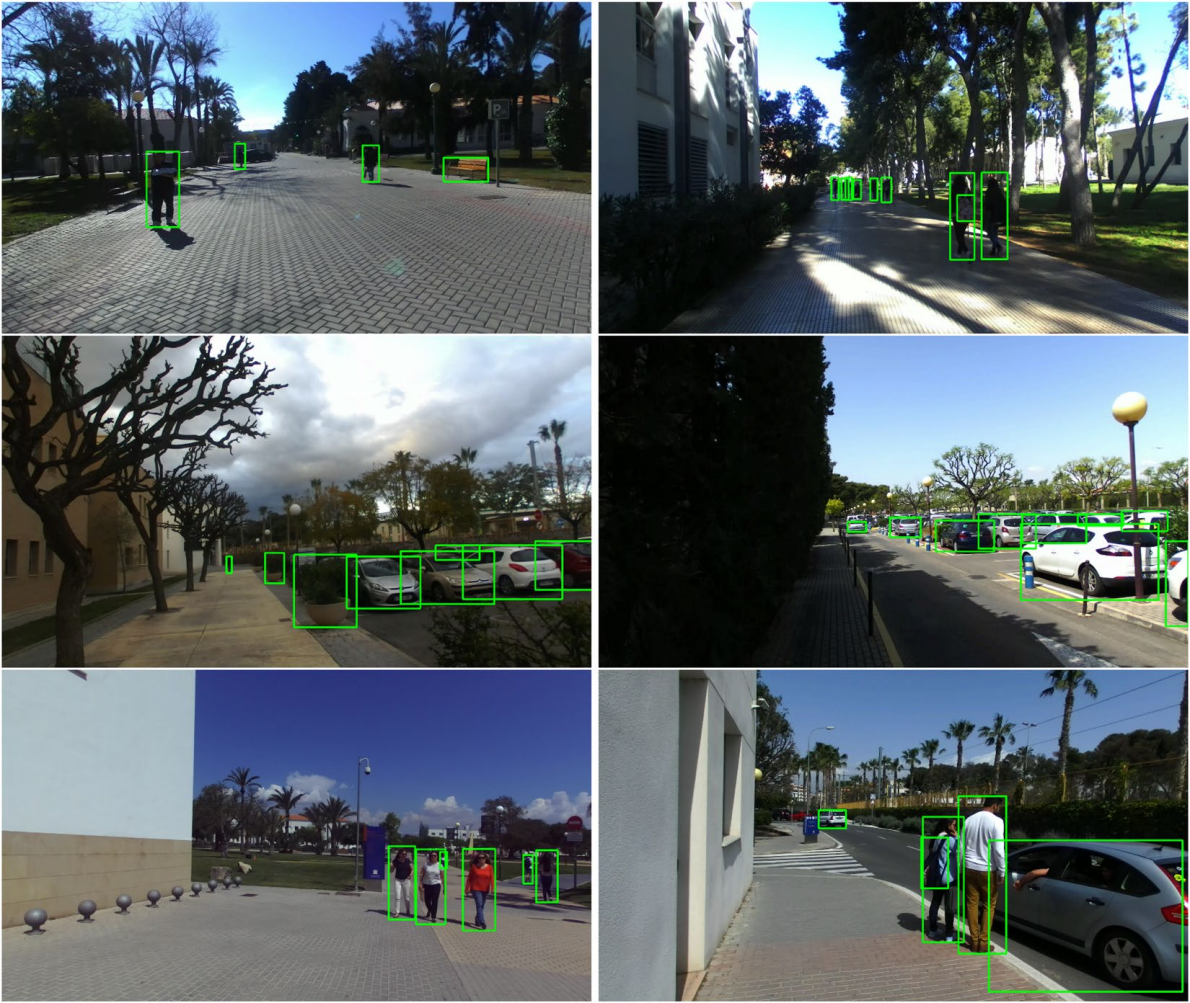
Results of the YOLOv3 architecture on random samples of the proposed dataset.

We chose YOLO over other state-of-the-art object detectors for several reasons. First, the base network runs at 45 frames per second with no batch processing on a Titan X GPU, which is considerably fast. In addition, YOLO achieves more than twice the mean average precision of other real-time systems. This is partially because it reasons globally about the input image when making predictions, so it sees the entire image during training and inference time. This causes the network to implicitly encode contextual information about classes as well as their visual appearance. Finally, it is worth noting that this approach learns generalizable representations of objects, so it is less likely to provide poor performance when applied to new domains or unexpected inputs. It is also worth noting that we did not train the system from scratch, but used a publicly available model already trained on the COCO^17^ dataset. Figure 2 shows the performance of the network on random samples of the proposed dataset.

We only considered a subset of the available labels provided by the CNN for the object counting, regrouping them as follows:

- Persons
- Vehicles with two wheels: bicycles and motorbikes
- Vehicles with four wheels: buses, trucks and cars
- Bags: suitcases, backpacks and handbags
- Benches

It should be mentioned that the rest of the labels provided by YOLOv3 (apple, kiwi, tie, train…) were not applicable. The final counting of objects using this method is shown in the *Technical Validation* section.

#### Pixel class counting procedure

In order to count the number of pixels that represent an object in each frame, we utilized the method proposed in^18^. This approach also explodes the convolutional neural network foundations explained earlier. However, in this case it is not used to classify and obtain the bounding box of an object from an input image, but to estimate segmentation masks. These segmentation masks can be translated to pixel-wise classifications, namely, the class of the object each pixel forms part of.

**Fig. 3:**
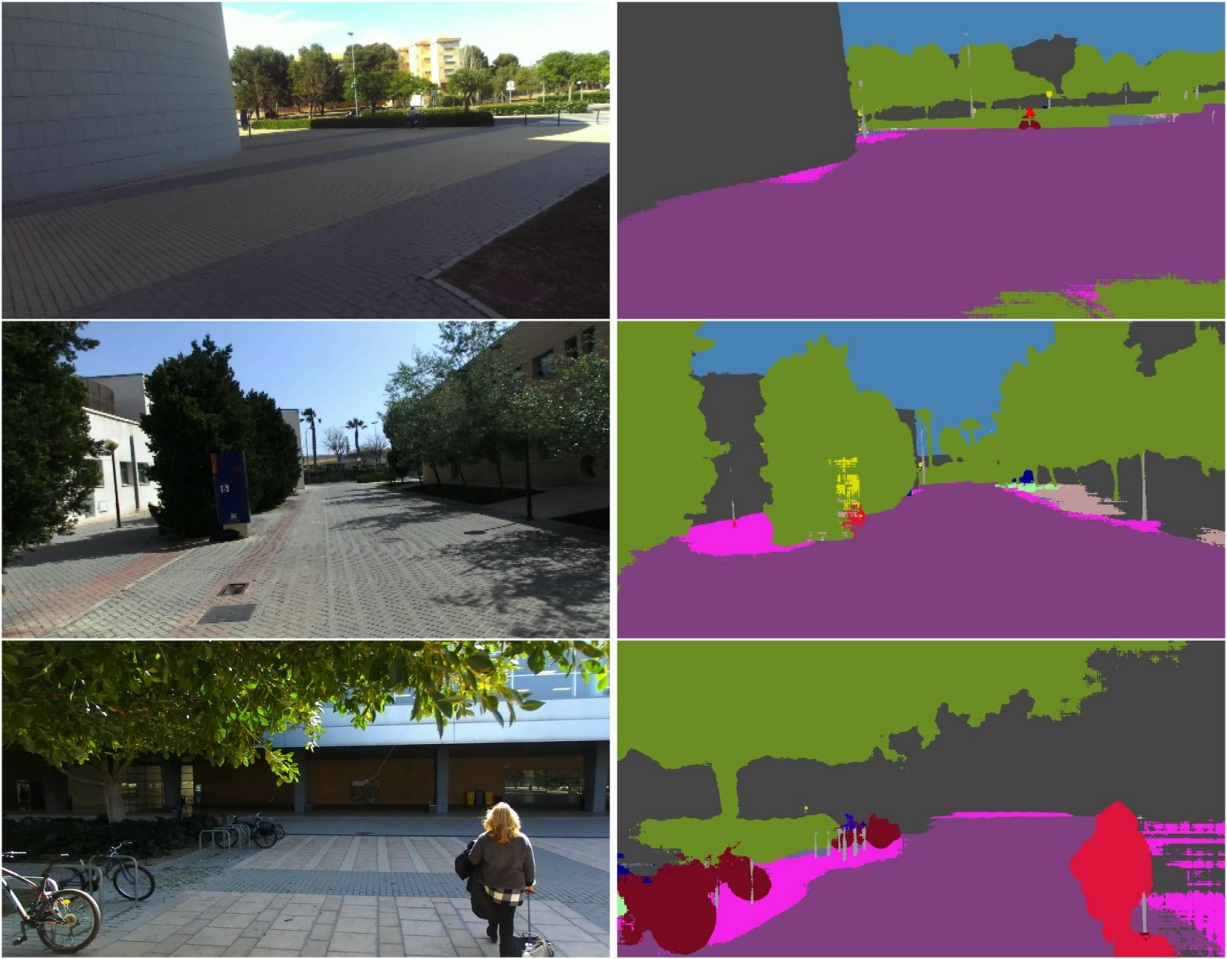
Estimated segmentation masks on random samples of the proposed dataset. Note that each color represents a different class.

There are several methods for estimating segmentation masks. Nonetheless, the chosen method provides state-of-the-art accuracy on several public datasets. Furthermore, it leverages the dilated up-convolutions concept, which allows it to avoid the gridding effect present in other approaches. This approach runs at about 2 seconds per inference step on a Titan X. Despite the method being rather slow, this is compensated for by the accuracy rate it provides. Finally, it is worth noting that the model we used was trained on the CityScapes^19^ dataset. Figure 3 shows the performance of this approach on random images from the proposed dataset.

We only considered a subset of the available labels provided by this method for the reported pixel class counting, also regrouping them as follows:

- Ground: roads and sidewalks
- Construction: buildings, walls and fences
- Vegetation
- Sky
- Persons: persons and riders
- Vehicles with two wheels: bicycles and motorcycles
- Vehicles with four wheels: buses, trucks and cars

It should be noted that the rest of the labels provided by this method (pole, traffic light, train…) were not applicable. The results obtained are shown in the *Technical Validation* section.

## Data Records

This section reviews different aspects of the data records we provide. We focus on the technical features of each one. All of the recorded data was stored in the Open Science Framework (OSF)^8^, more information about how the data is organized can be found at the *Organization section*.

### Stereo frames

As mentioned, the images of the dataset are provided by a ZED camera. This device features a stereo setup using dual lenses. It captures high-definition 3D video with a wide field of view and outputs left and right video streams in side-by-side format over USB 3.0. The left and right video frames are synchronized and streamed as a single uncompressed video frame.

Each frame of the dataset includes a pair of RGB images of the scene, captured at a resolution of 2208 × 1242 and at 15 frames per second. Parameters such as exposure, gain and white balance are automatically adjusted using the features of an on-board Image Signal Processor. The color images are stored in individual PNG files with no compression. Nonetheless, calibration parameters are included in the dataset to allow ad-hoc rectification and undistortion procedures. The method we used to model the distortion is thoroughly explained in^20^.

### Depth maps

For each stereo image pair in the dataset, the corresponding depth maps are also provided. As previously mentioned, we provide the depth maps generated by the ZED Semi-Global Matching and GC-Net algorithms. The depth maps store a distance value (Z) for each pixel (X, Y) in the image. The distance is a float value expressed in metric units and calculated from the back of the left eye of the camera to the scene. All the depth maps yield a 2208 × 1242 resolution and are stored in PNG with no compression.

### GPS data

The device used to collect GPS data is the GlobalSat ND-105C. This device is able to provide GPS localization with an error of less than 3 meters.

Each pack of left and right RGB and depth maps frames is GPS-tagged. As mentioned before, the camera captures images at 15 fps, but the GPS tracker is only able to provide one position per second. In order to synchronize both devices, the system clock timestamp is used so that each frame is assigned to the nearest in time GPS data. GPS data includes latitude and longitude, which are expressed in decimal degrees unit.

The accuracy of the data was qualitatively evaluated after each scene recording by projecting the collected GPS data on a satellite map. A GPS recording of a random sequence can be seen in the Fig. 1.

### Positional tracking

Positional tracking is the ability of a device to estimate the position of observers relative to the world around them. This is used to track the movement of a camera or user in 3D space with six degrees of freedom. The ZED camera provides positional tracking. It features a vision-based algorithm that computes rotation and translation between two consecutive frames. The exact method to do this is based on a custom implementation that was not made public, so no further details are known. The first frame of each sequence is used as the fixed world coordinate frame and the poses are given for the left eye of the device. We provide a translation vector that represents the displacement between the current camera position and the reference coordinate frame in metric units and the three rotation Euler angles obtained using the Rodrigues^21^ formula. In addition, we also provide the same rotation and translation vectors in a transformation matrix that encodes both data and is more suitable to work with for some applications.

### Organization

The dataset is divided into folders. Each folder contains the data for a particular sequence. Inside each folder, there is a log file, a manifest file and another folder with the actual RGB color pairs and the corresponding depth maps. The directory tree of the dataset is shown in the Supplementary Material section, Fig. 2.

#### Log file

The TXT file named “log” (log.txt) stores the camera settings. This data was obtained using the ZED API. A log file is provided for each sequence. The information provided is listed below:

For each camera:

- Optical center along x axis (pixels)
- Optical center along y axis (pixels)
- Focal length along x axis (pixels)
- Focal length along y axis (pixels)
- Vertical field of view after stereo rectification (angle in degrees)
- Horizontal field of view after stereo rectification (angle in degrees)
- Diagonal field of view after stereo rectification (angle in degrees)
- Distortion factor of the right cam before calibration (as described in^20^)
- Distortion factor of the right cam after calibration (as described in^20^)

For each sequence:

- Confidence threshold
- Depth min and max range values (millimeters)
- Resolution of the images (pixels)
- Camera FPS
- Frame count

#### Manifest file

The “manifest” file (complete.json) packs the core information for each sequence. The information provided is listed below:

- Filename of the left color image
- Filename of the right color image
- Filename of the depth map as provided by the SGBM algorithm
- Filename of the depth map provided by the GC-Net method
- Translation matrix (3 × 1)
- Orientation matrix (3 × 1)
- M matrix (4 × 4) which contains the rotation and translation
- Timestamp (ms)

### Train, test and validation splits and evaluation metrics

We suggest a train, validation and test split. In this way, a set of sequences is used for testing, a different set for validation, and the remaining sequences for training. The proposed splits are shown in Table 3. We adopted this methodology because it ensures that the generalization capabilities of the approaches are properly tested, as the test and validation splits include visual features that the training set does not have.

**Table 3:** Train, Validation and Test splits with the sequences it comprises and the number of total frames.

Splits	Sequences	Frames
Train	Alumns Help Desk, Lecture Rooms I, Lecture Rooms II, Library, Biotechnology, Sciences II, Sciences III, Sciences IV, Sciences V, Social Sciences, Club I, Economics, Multipurpose II-III, Nursery, EPS1, EPS4, German Bernacer, University, Rectorship, University 12, University 13, Optics	110835
Validation	Shopping Center, Science I, Club II, Law, EPS2, EPS3, Philosophy I, Philosophy II-III, Garden	48688
Test	Multipurpose I, Control Tower	5842

Additionally we introduce a subset of 676 pairs of RGB Stereo images and their respective depth, which we extracted randomly from the entire dataset. This given test set is introduced to make comparability possible between the different methods trained with the dataset.

To properly evaluate this dataset, we next propose a protocol to be followed. Reporting the proposed performance metric ensures reproducibility and comparability results of any method that uses this dataset.

The following equation must be fulfilled to carry out the computation of the proposed depth error metric:

$$Error=\mathop{\sum }\limits_{j=0}^{M}\,\mathop{\sum }\limits_{i=0}^{N}| {p}_{ij}-{p}_{ij}^{GT}| $$

Where:

- M = number of test images
- N = number of pixels of an image
- The valid pixels (*p*_*ij*_) are those where $i\,\,\epsilon \,\,{\mathbb{N}}$ and *i* ≠0 and the valid distance range is between 0.5 and 20 meters.
- ${p}_{ij}^{GT}$ = The valid pixels in the ground-truth depth maps.

## Technical Validation

This section presents the qualitative and quantitative analysis conducted to support the reliability of the dataset. It also provides additional information related to the quality of the dataset, such as number of objects, representativeness of the pixels in the images and depths distribution.

### Semantic Variability

**Fig. 4:**
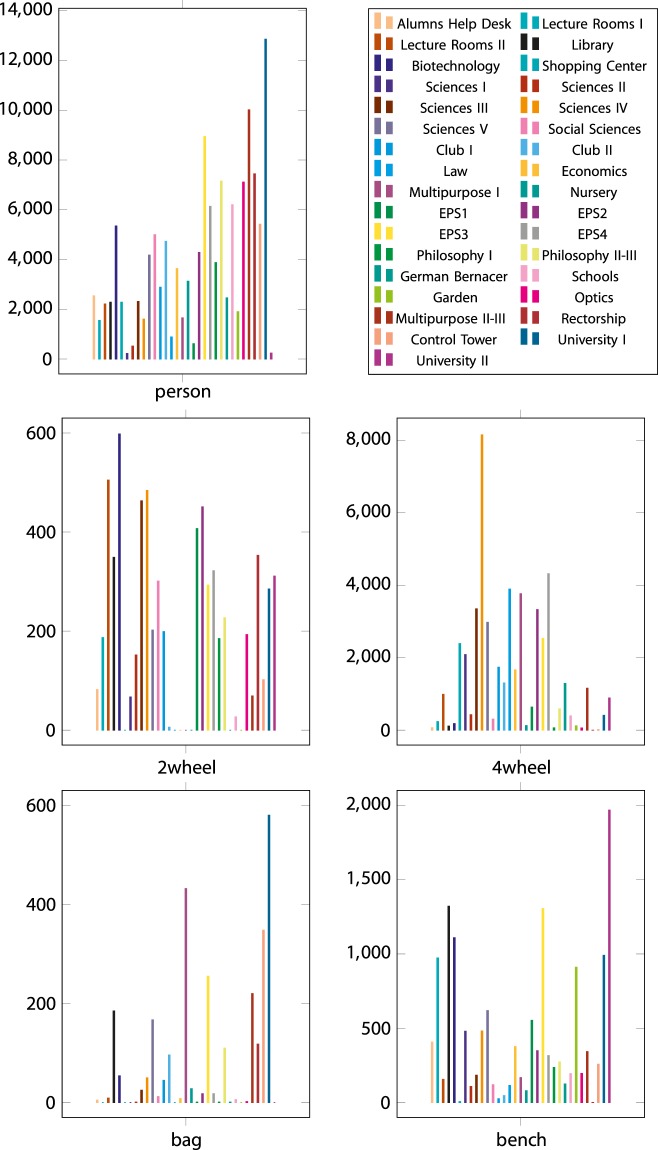
Counting of interesting objects for each sequence of the proposed dataset. The values are expressed in absolute number of objects.

**Fig. 5:**
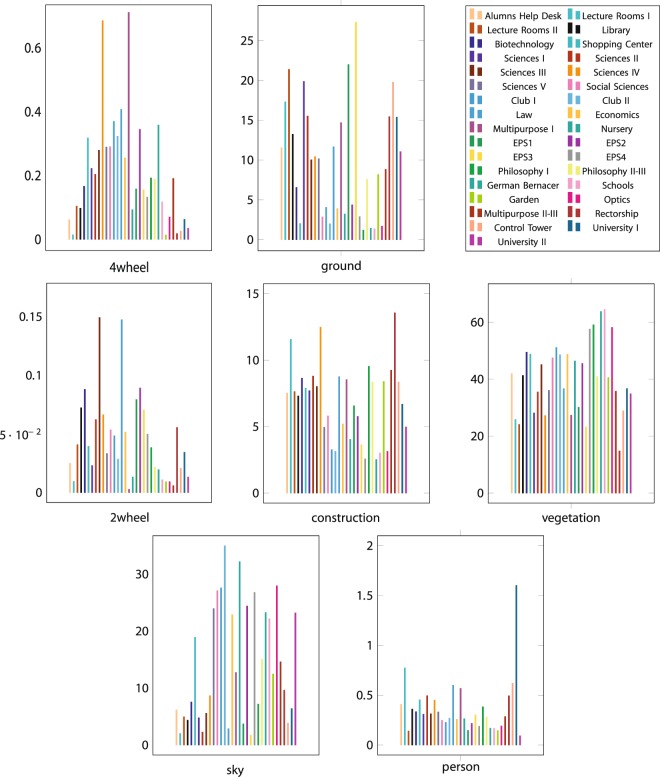
Percentage of the quantity of pixels that belong to a certain class in each sequence of the proposed dataset.

In order to provide more insight into the content of the dataset, we counted a number of interesting objects and the percentage of frame occupied for a semantic class along the sequences. This information is shown in Figs 4 and 5.

The objects and pixel-wise classifications were extracted as explained in the Object Counting Procedure and Pixel Class Counting Procedure subsections. This information is provided in order to allow the users of the proposed dataset to properly choose which sequences suit the application they are looking for. For instance, if they focused their algorithm in persons, they must evaluate it with the University I sequence because it features a large number of persons, rather than University II, which has a smaller number of persons.

### Semi-global matching and GC-Net algorithms

**Fig. 6:**
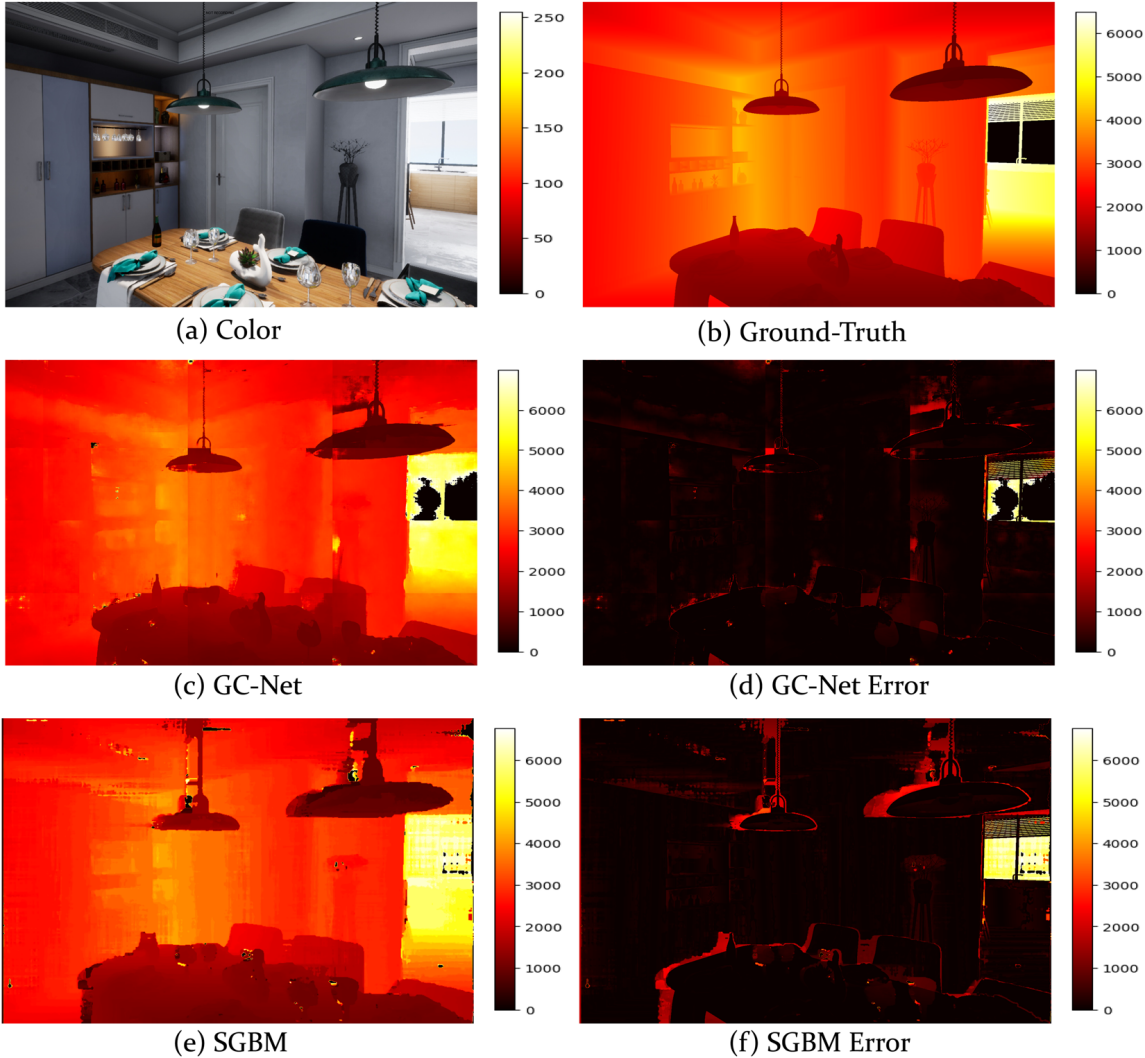
Results of the Semi-Global Matching algorithm and the GC-Net algorithm using the synthetic UnrealROX dataset (depth images are in millimeters).

The results of the Semi-Global Matching and GC-Net algorithms are shown in Fig. 6.

Mean error of the GC-Net algorithm: 180.52 mm.

Mean error of the SGBM algorithm: 493.93 mm.

Note that the images used to compare the algorithms were extracted from the UnrealROX dataset^22^. This is a synthetic dataset that features photorealistic images. We selected it because not only does it provide realistic visual features, but the scenes are arranged in a realistic manner. Moreover, it provides perfect depth maps, so it is appropriate for conductingsuitable for getting good results.

Figure 6 is intended to show the different depth data provided by the UASOL dataset. In our dataset, we provide both types of data: SGBM (Zed) and GCNet, because they are both used as baseline for Stereo, one is based on traditional vision, with a handmade match function and the GCNet algorithm is based on deep learning methods, leveraging a network able to learn how to find matches. This gives the data to an author who wants to compare their own network with one of these baselines, without needing to train any model, simplifying the comparison process for anyone wanting to use the dataset.

As can be seen in Fig. 6, images a and b show the color image and the corresponding ground-truth. Images c and e are the predictions obtained using the GC-Net algorithm and the Semi-Global matching algorithm, and Images d and f show the difference between the original ground-truth and the predicted depth maps.

The results presented show that, on the one hand, the GC-Net algorithm works more effectively than the SGBM algorithm on ambiguous areas, but, on the other hand, it performs poorly on areas with low texture where the SGBM algorithm yields good results. The SGBM algorithm works with higher precision on elements at a greater distance (about 8 meters), enhancing accuracy to the predicted distances. The GC-Net algorithm archieves more precise distance results at elements at lower distances (8 meters).

**Fig. 7:**
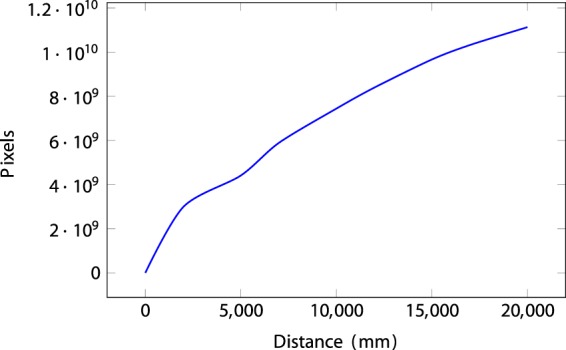
Cumulative plot of the depth values contained in the scene “Alumns Help Desk”.

Additional results of the algorithms are in the Supplementary Material section, Figs 7, 8, 9 and 10.

### More variability and generalization features

As stated in the Procedure section, the capture setup is mounted on a person’s head. Four-4 individuals and 38 different buildings’ surroundings were involved in the dataset acquisition. It also features different weather conditions such as cloudy, sunny and partly cloudy. Different moments of the day such as morning, afternoon and evening are also depicted. The consideration of these cases ensures high variability of the data, which will challenge the generalization capabilities of the algorithms using this dataset.

Table 4 shows the distribution of the scenes based on the meteorological conditions during the recording. As mentioned, it is divided into sunny, cloudy and partially cloudy.

**Table 4:** Weather conditions in the different sequences of the dataset.

Weather Condition	Sequences
Sun	Alumns Help Desk, Lecture Room I, Lecture Room II, Library, Biotechnology, Sciences II, Sciences III, Multipurpose I, Multipurpose II-III, Nursery, EPS1, EPS2, German Bernacer, Rectorship, University I, Control Tower, Garden, Philosophy I, Philosophy II-III
Partially cloudy	Shopping Center, Sciences I, Club II, Law, EPS3, EPS4, Optics, Sciences IV, Social Sciences, Club I, Economics
Cloudy	Sciences V, University I, University Institute

As shown in Table 4, most of the sequences were recorded with sunny weather conditions, but there are also partially cloudy and cloudy weather sequences. In this case, cloudy means close to rain. It can therefore be said that the dataset contains sequences with different lighting conditions.

Table 5 shows the recording periods of the sequences so that the different brightness variations of the frames can be taken into account.

**Table 5:** Recording times for the different sequences of the dataset.

Recording Schedule	Sequences
Morning (08.00 h–14.00 h)	Alumns Help Desk, Lecture Room I, Library, Biotechnology, Sciences I, Sciences II, Sciences III, Science IV, Law, Economics, Multipurpose I, Multipurpose II-III, EPS2, EPS3, EPS4, German Bernacer, University I, Optics, Control Tower, Philosophy I, Philosophy II-III, University II
Afternoon (14.00 h–20.00 h)	Lecture Room II, Shopping Center, Sciences V, Club 1, Club 2, Nursery, EPS1, German Bernacer, Garden, Social Sciences, Rectorship

All the times range from 8.00 a.m. to 8.00 p.m. The timings are in Spanish summer time zone. This feature provides the images with different sunlight locations throughout the day, thus yielding, different lightning conditions.

Figure 7 shows a cumulative line plot of the depth values contained in each frame of a sequence (Alumns Help Desk). As can be seen, a large quantity of pixels are located at a distance of between 0.5 and 2 meters, although the distribution of depths in the sequence is quite uniform. This pattern is consistent through all the scenes.

Additional results are in the Supplementary Material section, Figs 3, 4, 5 and 6.

### Baseline

As baseline we present the following results from the monocular depth algorithm by Iro Laina^23^, the same network as that trained by us (the original algorithm does not provide code to train it), and the network trained using the UASOL dataset, the results are shown in Table 6.

**Table 6:** Baseline for the dataset.

Test Set	Architecture and Model	MRelE	RMSE
UASOL	Ref.^23^ (publicly available model)	0.753	8.119
	Ref.^23^ (trained on the UASOL dataset by us)	0.326	4.4134

The results for the tested models can be found in the Supplementary material section in Fig. 11. As can be seen, the original network shows problems in predicting the correct ground structure and in handling different natural conditions such as shadows and direct sunlight. Training the models with the dataset enhances the predictions in thede scenarios, making the network more generalizable.

### Future work

As future work we will focus on the application of slam techniques across the scenes for a complete 3D reconstruction of the whole dataset, giving the possibility of creating a virtual tour of the university.

## Supplementary Information

### ISA-Tab metadata file

Download metadata file

### Supplementary information

Supplementary Material

## Data Availability

We uploaded a selection of Python scripts to the public repository of the project. First, the *read data main*.*py* is in charge of taking a raw ZED recording file SVO and saving the left and right color images, and the corresponding depth map. It also saves the manifest JSON and the log files. This script is largely based on the ZED sample script and needs GPU capabilities to compute the depth maps. It also needs the ZED API v2.3.3. Then, the *data*.*py* is a sample script that takes a manifest JSON file and loads the sequence into python variables. We used the script named *gps*.*py* to connect to the GPS device. This script queries the current geolocalization in an infinite loop. When closed, it saves the data to a JSON file. Next, the script *gps2csv*.*py* takes a JSON file generated by the previous script and dumps the data to a comma-separated text file. This file is intended to be uploaded to a GPS Visualizer. in order to visually inspect the accuracy of the GPS records.
