# Supplementary material for: UASOL, a large-scale high-resolution outdoor stereo dataset
Source: Sci Data. 2019 Aug 29;6:162. doi: 10.1038/s41597-019-0168-5 (PMC6715739; doi:10.1038/s41597-019-0168-5)
Supplement: Supplementary file 2 — Supplementary Material [file 41597_2019_168_MOESM2_ESM.pdf]

Supplementary Material

In this section you will find the additional images to the previously exposed paper. Additionally we have included more examples of results obtained by the different algorithms used for the work.

The information included in this section is:

Supplementary Table. 1: Table of contents.

Figures	**Information**	**Pages**
1	Camera depth error (Distance vs Error)	2
2	Directory tree.	3
3 to 6	Accumulative plot of the depth values in different sequences.	4 to 6
7	Results of the SGBM and GC-Net algorithm.	7 to 10
3 to 6	Errors from the baseline models.	11

Accuracy of the ZED Camera

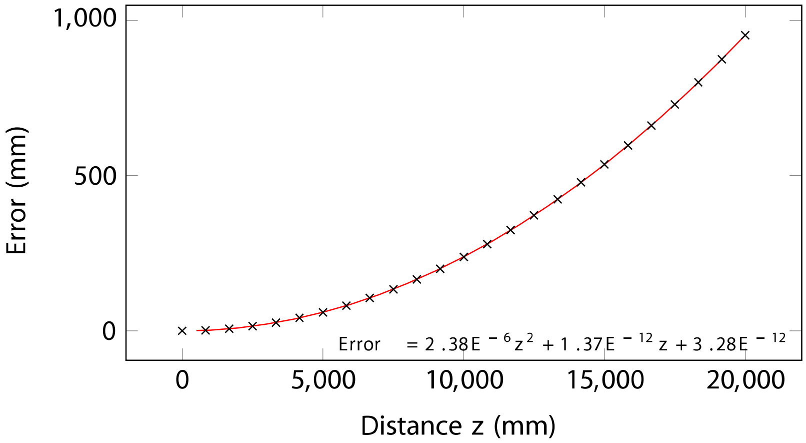

Supplementary Figure. 1: Depth Error from the ZED Camera, Distance vs Error (both units in meters)

SCIENTIFIC DATA

Overleaf

Directory Tree

```
│ Alumns Help Desk (Sequence 1)
│   ├── log.txt (Log file)
│   ├── complete.json (Manifest file)
│   └── Images
│         ├── img_0_depth.png (depth map 0 as generated by the ZED Stereo
│         │   algorithm)
│         ├── img_Y_depth.png (depth map Y as generated by the ZED Stereo
│         │   algorithm)
│         ├── img_left0_color.png (left lense image 0)
│         ├── img_leftY_color.png (left lense image Y)
│         ├── img_right0_color.png (right lense image 0)
│         └── img_rightY_color.png (right lense image Y)
├── Alumns Help Desk GC-Net (Sequence 1)
│   ├── img_0_depth.png (depth map Y as generated by the GC-Net method)
│   └── img_Y_depth.png (depth map Y as generated by the GC-Net method)
└── name (Sequence N)
    └── ...
```

Supplementary Figure. 2: The directory tree of the dataset.

Note: the images are compressed in .ZIP files.

Cumulative line plot of the depth values

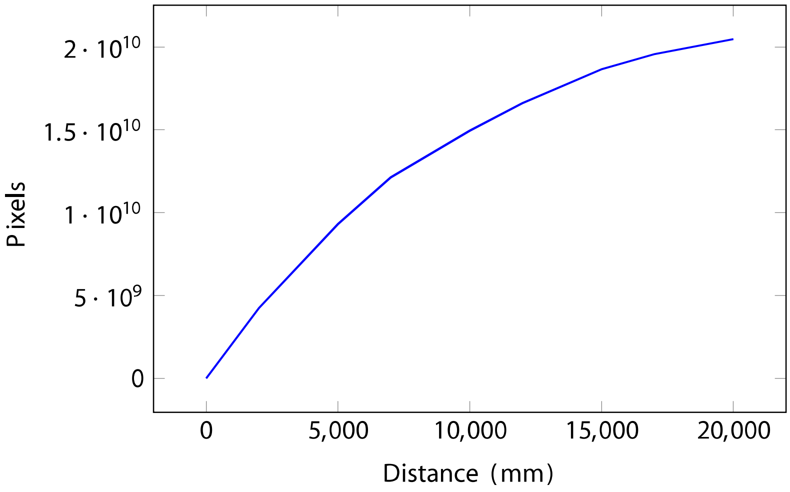

Supplementary Figure. 3: This figure shows the accumulative plot of the depth values contained in the scene "Lecture Rooms I".

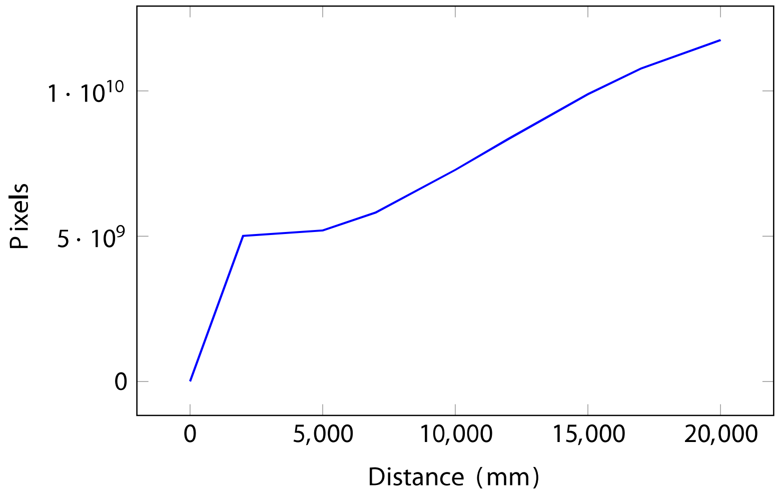

Supplementary Figure. 4: This figure shows the accumulative plot of the depth values contained in the scene “Biotechnology”.

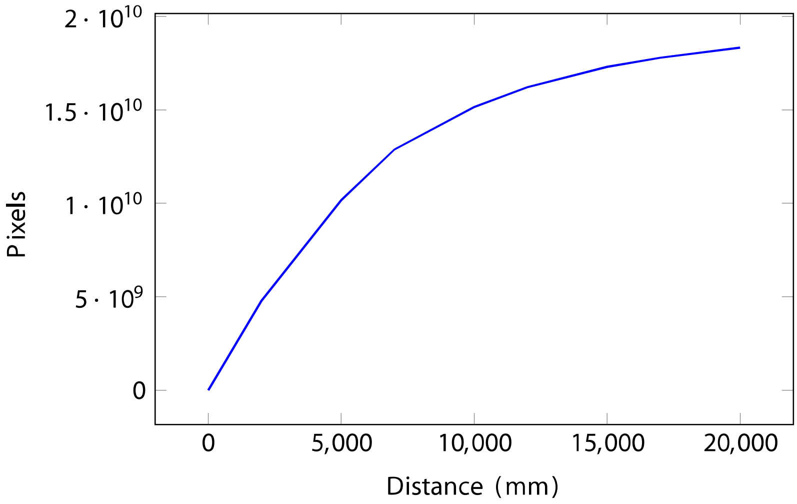

Supplementary Figure. 5: This figure shows the accumulative plot of the depth values contained in the scene “Science 1”.

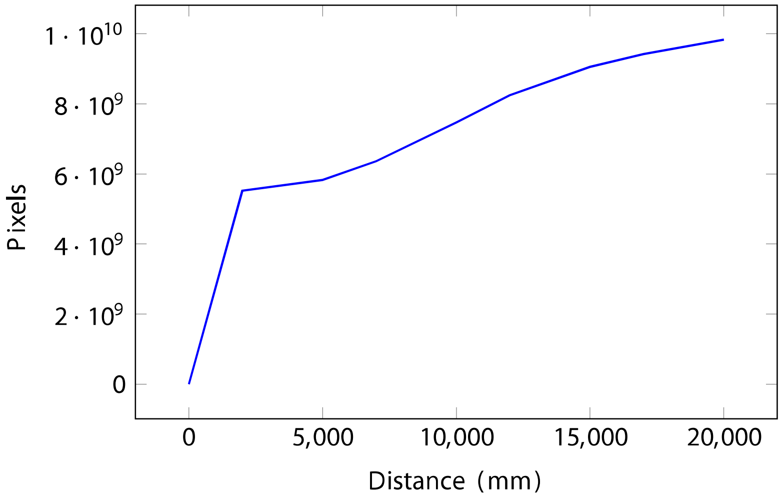

Supplementary Figure. 6: This figure shows the accumulative plot of the depth values contained in the scene “Club 1”.

SCIENTIFIC DATA

Semi-Global Matching and GC-Net algorithms

Additional predictions of the Semi-Global Matching algorithm and the GC-Net Algorithm with their corresponding mean error.

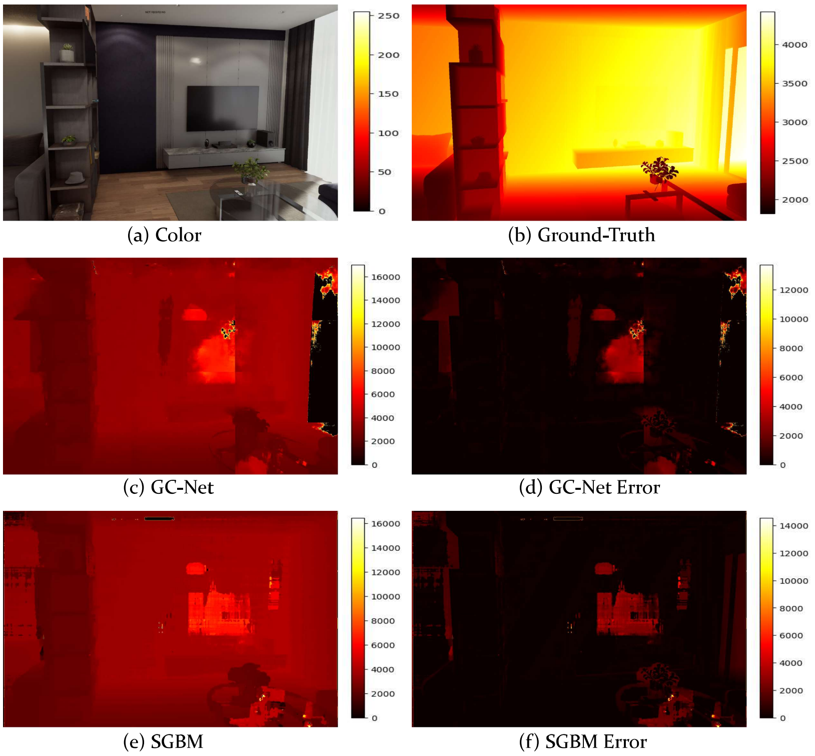

Supplementary Figure. 7: Results of the Semi-Global Matching algorithm and the GC-Net algorithm using the synthetic UnrealROX dataset

Mean error of the GC-Net algorithm: 248.36 mm
Mean error of the SGBM algorithm: 560.24 mm

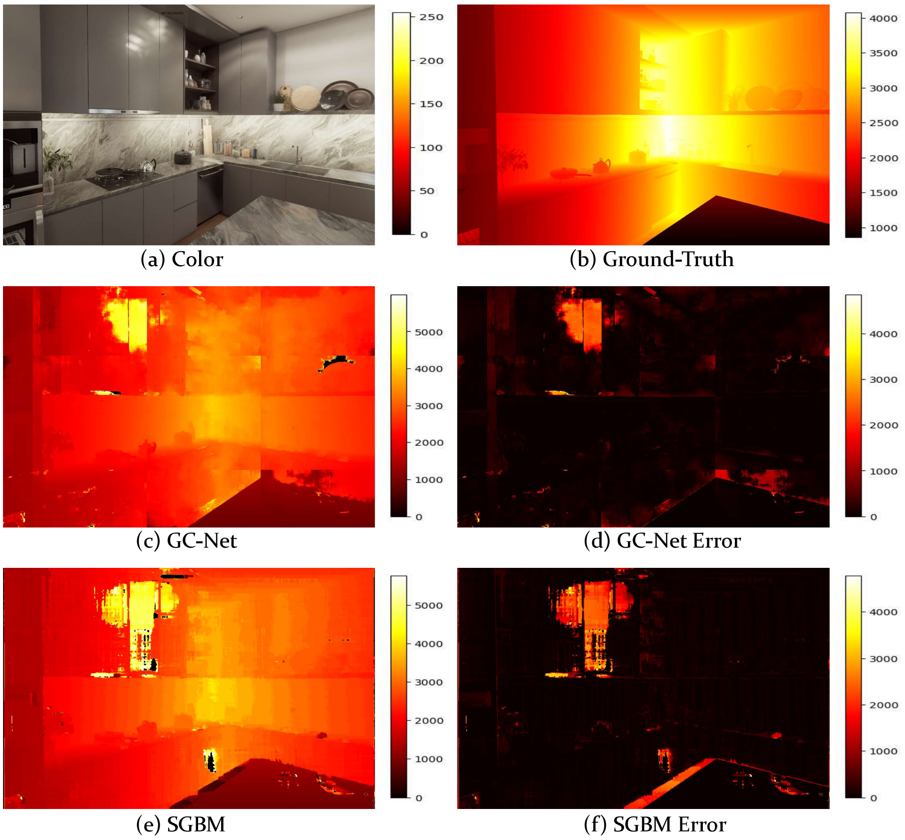

(a) Color (b) Ground-Truth (c) GC-Net (d) GC-Net Error (e) SGBM (f) SGBM Error

Supplementary Figure. 8: Results of the Semi-Global Matching algorithm and the GC-Net algorithm using the synthetic UnrealROX dataset

Mean error of the GC-Net algorithm: 123.12 mm
Mean error of the SGBM algorithm: 244.33 mm

SCIENTIFIC DATA

Overleaf

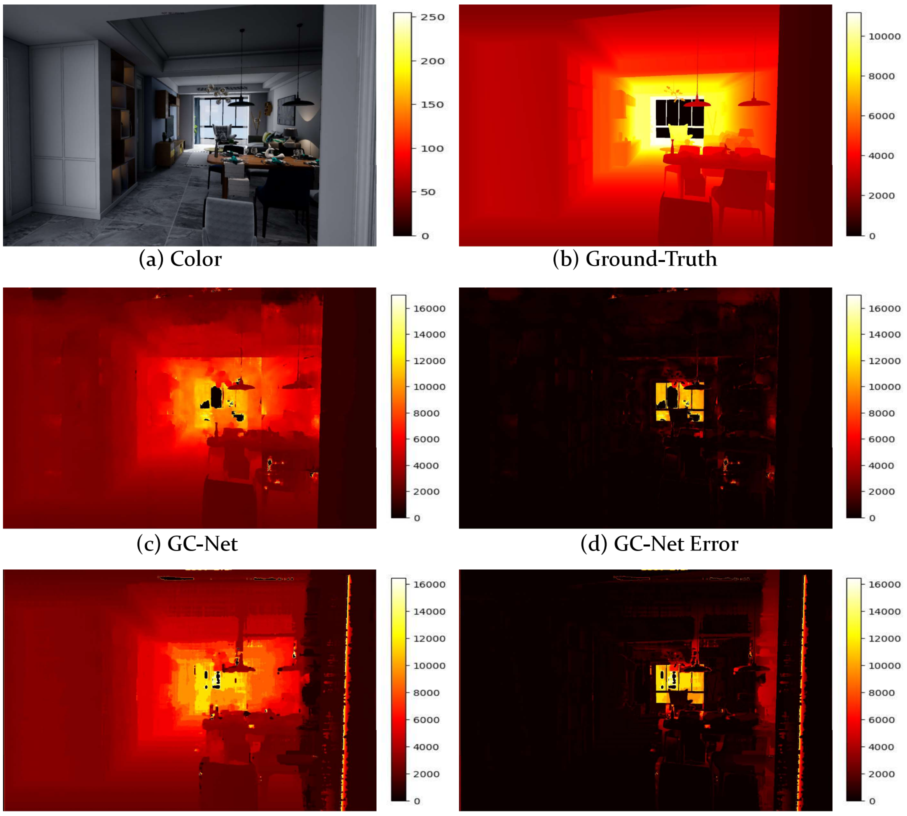

(a) Color (b) Ground-Truth

(c) GC-Net (d) GC-Net Error

(e) SGBM (f) SGBM Error

Supplementary Figure. 9: Results of the Semi-Global Matching algorithm and the GC-Net algorithm using the synthetic UnrealROX dataset

Mean error of the GC-Net algorithm: 281.18 mm
Mean error of the SGBM algorithm: 742.34 mm

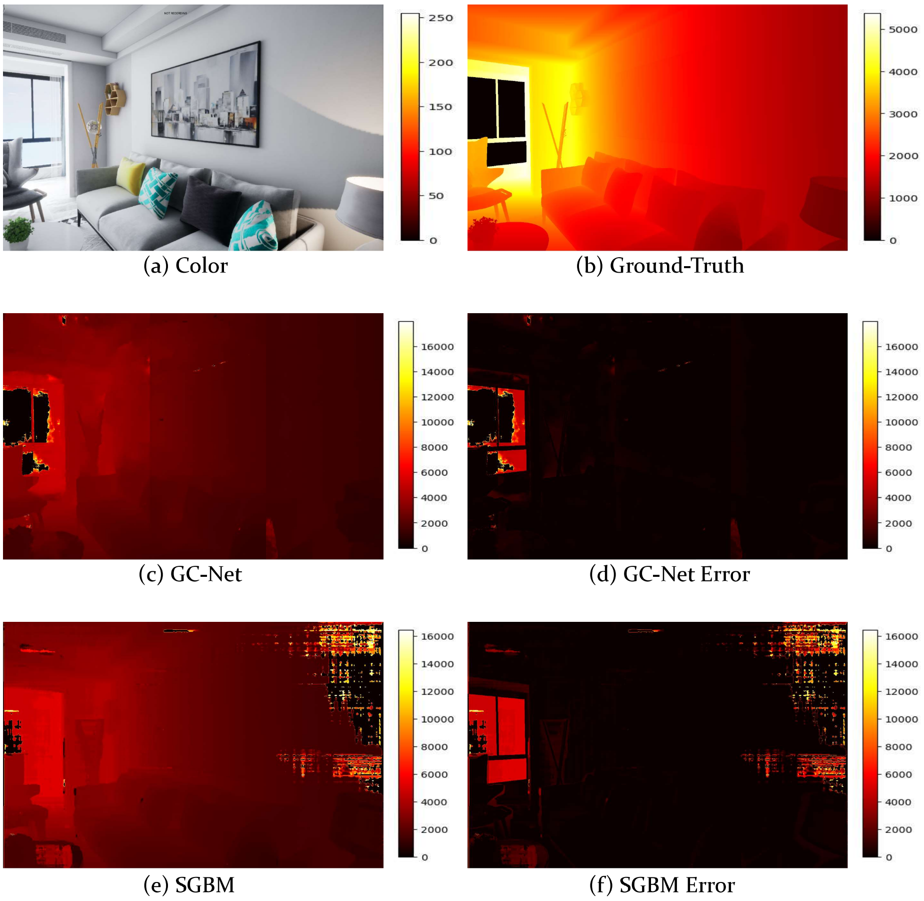

(a) Color (b) Ground-Truth
(c) GC-Net (d) GC-Net Error
(e) SGBM (f) SGBM Error

Supplementary Figure. 10: Results of the Semi-Global Matching algorithm and the GC-Net algorithm using the synthetic UnrealROX dataset

Mean error of the GC-Net algorithm: 234.19 mm
Mean error of the SGBM algorithm: 818.65 mm

Baseline

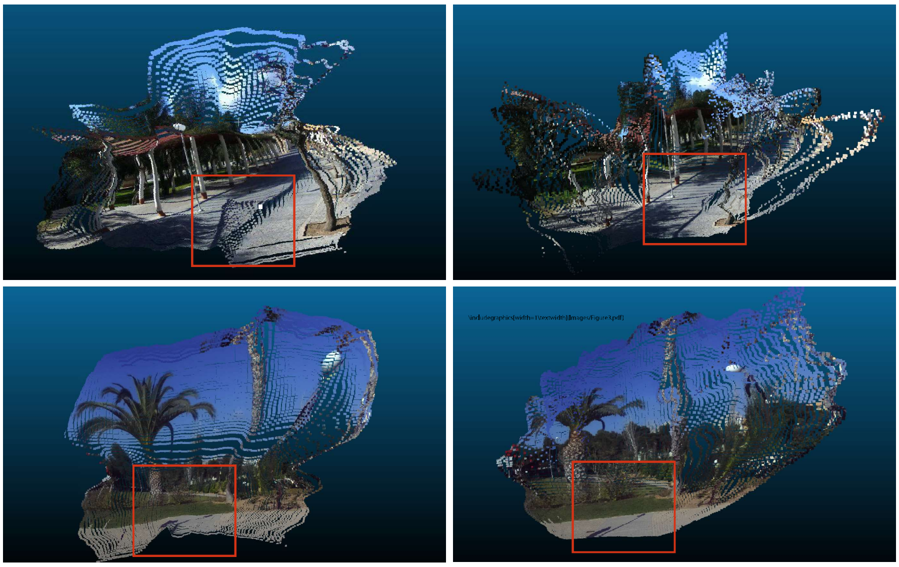

Supplementary Figure. 11: Errors from the Baseline model.
